# Practical Essays on Medical Education and the Medical Profession in the United States

**Published:** 1829

**Authors:** 


WESTERN JOURNAL

OF THE

MEDICAL & PHYSICAL SCIENCES.

APRIL, MAY, & JUNE, 1829.

### ESSAYS AND CASES.

Art. I.—*Practical Essays on Medical Education and the Medical Profession, in the United States.*—By the Editor.

### ESSAY I.

### Selection and Preparatory Education of Pupils.

Of the various occupations in society, scarcely one requires greater talent and attainment, than the medical profession. This is especially true in the United States, where almost every practitioner must be at the same time, physician, surgeon and apothecary. Obvious as this proposition is to many, its truth, unfortunately, is not generally perceived by those who are about to dedicate their sons to the profession,—in other words, by the persons who above all others should feel and acknowledge its reality. Hence it results, that the ranks of the profession are in a great degree filled up with recruits, who are deficient, either in abilities or acquirements (too often indeed in both,) who thus doom it to a mediocrity, incompatible both with its nature and objects. Other causes contribute to its degradation; but this I am persuaded is one of the most frequent and most difficult to obviate. Still much might be done, if those who have the power, would open their eyes to the evil, and exert their influence in its suppression.

Few of those who are put to the study of medicine, can be aware of the magnitude of the undertaking, or of the insufficiency of their capacity and preparation; for the obvious reason, that they are in general young and inexperienced. There are, however, two classes of persons, who might be expected to judge more correctly, and have much in their power. I refer to parents and physicians, both of whom rather than our sons, should feel responsible to society on this subject; and to them I beg leave respectfully to address myself.

In the selection of boys for the study of medicine, many circumstances entirely disconnected with their fitness, too often exert a dominant influence; when their sway should be subordinate, or even regarded as entirely inadmissible. A neighbouring physician wants a student to reside in his office; or one son of the family is thought too weakly to labour on the farm or in the work shop; he is indolent and averse to bodily exertion; or addicted to study, but too stupid for the Bar, or too immoral for the Pulpit; the parents wish to have one gentleman in the family, and a *doctor* is a *gentleman*:—these and many other extraneous considerations, not unfrequently decide the choice and swell the numbers, while they impair the character of the profession.

Both parents and physicians should know, that boys of feeble frame and unsound constitution, cannot endure study, and are best strengthened by hard labour; they should not, indeed, ever be put to the learned professions, unless they chance to possess extraordinary genius. Every physician must have seen many, who dragged out the whole period of their brief and reluctant pupilage, with dyspepsia, a pain in the breast, or hypochondraism; conditions which will preclude all intense and successful application; or render it the cause of some other distressing malady, which terminates in premature death.

But it is not sufficient that boys selected for the study of medicine, should have good constitutions; they ought, equally, to be endowed with vigorous and inquiring minds. Without these, whatever may be the appearances of success, they must at last make incompetent physicians. It is especially and indispensably necessary, that they possess, in a high degree, the faculties of observation and judgment; without which, they can neither comprehend the principles of the science, nor apply them correctly in the treatment of diseases. Notwithstanding this obvious fact, hundreds are put to the study of medicine, whose utmost grasp of intellect never encompasses the first principles of the science. As a matter of course, they slur over every difficult proposition, and afterwards grope their way for forty years, unconsciously committing 'sins of omission or commission,' throughout the whole of that long period. It is in vain to rely on society to correct this great evil, by discriminating among the candidates for their confidence; for the knowledge necessary to a correct selection, does not exist among them. In the other learned professions, this species of empyricism cannot be practised. The incompetent Divine, at most but occupies the place of an abler teacher, and the superficial Lawyer, is either driven from the Bar, by the exposure of his errors, or they are rendered harmless, by the skill of competent associates. But the physician, who has passed through the usual forms of a professional education without the capacity to improve by his opportunities, is presumed by the people, to be qualified for every emergency; and sometimes even preferred to the ablest practitioners.

The student of medicine should not only be of sound understanding, but imbued with ambition. A mere love of knowledge is not to be trusted to, for the greatest lovers of knowledge, are not unfrequently deficient in executive talents, and go on acquiring without learning how to appropriate. Let parents, therefore, not be misled by the signs which indicate a fondness for study, unless the desire involves a feeling of emulation. A thirst for fame, is indeed a safer reliance, than a taste for learning; as it generates those executive efforts, which are indispensable to the successful practice of the profession.

Further, the temperament of the youth, should be that of industry and perseverance; without which he will balk at every difficulty, and require close watching through all stages of his pupilage. An indolent or irresolute student, whatever may be his talents, can never figure as a physician, and should without delay be apprenticed to some vocation—in which the destruction of limbs and life will not be the inevitable consequence of idleness and discouragement.

Finally, parents who are too poor to afford their sons the necessary opportunities, should not aim at making them physicians. If we now and then see one, whose talents, ambition and enterprize, have enabled him to acquire distinction, in despite of every obstacle, we meet with many more, who all their lives remain unfinished and imperfect, from the want of adequate time and opportunities, while engaged in their professional studies. I am the more disposed to insist on these truths, because so many fathers are ignorant of what is really necessary to make their sons good physicians; and place them to the study of physic before they have accurately counted the cost. Of all the causes which impede the progress of medicine in the United States, not one is more operative than this. The amiable vanity in which it originates, can scarcely be condemned; but parents should be admonished to look at the consequences of such an indulgence of feeling. Under the most limited opportunities, a son can make acquirements that may satisfy a fond father, who knows but little of the extent and complexity of the medical sciences; to be prepared, however, for the various exigencies of the profession, is a much more difficult affair. Paternal affection may blind us to the errors of our sons, but cannot obviate their prejudicial influence on society.

Having briefly considered the moral and physical qualities which fit young men for the study of medicine, I come now to say, that a majority of those who are selected for that purpose, are deficient in one or both classes of requisites. Let us enquire into the principal causes of this state of things —so unfavourable to the dignity of the profession, and prejudicial to the interests of humanity.

The current opinion, that men of slender talents are competent to the practice of physic, is obviously a great cause why so many feeble minded boys are dedicated to its study.

There never existed a time, when this opinion was well founded. In past ages, when medicine could not claim a place in the ranks of philosophy, it was still a science of observation, and called for an acute and discriminating mind. Though it could not rise above the grade of an experimental art, it never sunk to a trade, the results of which were purely accidental, but when it fell into the hands of the imbecile or the unprincipled. At the present time, when the intricacies of the human structure have been unravelled, and many of its functions are understood; when the accumulated experience of centuries has developed numerous general truths, and the spirit of inductive philosophy has arranged them into a science—imperfect it is true, but still a science—when chemistry, natural philosophy, botany and natural history, have become essential preliminary branches, nothing can be more adventurous, than to engage in the study of the profession without a logical and comprehensive mind.

Another cause of the evil which we deprecate, is the liberal proportion of inferior men, who unfortunately belong to the profession, and so often succeed in acquiring business and popularity. An observation of this fact, not a little mortifying to the more talented members of the profession, encourages parents to set apart for the study of medicine, those sons who are least remarkable for strength of mind; reversing, the better class, for pursuits which in their opinion require more vigour of intellect, or promise greater distinction. Thus a degraded state of the profession is made to perpetuate itself, by influencing, not only the ignorant but the well informed; for when a father, who has a just estimate of the strong talents necessary to the study and practice of physic, apprehends that a son, of whose genius he is proud, may, on entering the arena, meet with many whose emulation is vitiated with envy; who labour to please rather than preserve their patients; or whose skill consists more in the arts of imposture than of cure; it is not unnatural for him to shrink from the contemplation of the contest.

The root of this great evil is planted in society itself. Some persons are too dull to distinguish among the members of the profession, others allow themselves to be captivated by pleasant manners, and not a few call for *cheap doctoring*, all of which tend directly to elevate false pretension and depress real merit.

But there are causes which attract, as well as repel, young men of genius from the profession.

It will not be denied, that no passion is more intense and universal, than the love of gain. If not the first to be developed, it is the last to be extinguished; and, taken in the aggregate, perhaps no other exercises so much sway over the course and conduct of man. In most parts of the United States, the practice of medicine offers but little to gratify this desire, in comparison with commercial pursuits; and hence we find, that *they* exert an influence, which draws into the vortex of trade, no inconsiderable part of the talents and enterprise of the nation.

Strong, however, as the attractions of commerce unquestionably are, their influence is trifling, compared with those of law and politics. In no other country is the union of those two sciences so intimate, as in the United States. The regular course of promotion is from the county court house to the capital of the state, thence to the halls of congress, and, finally to the presidential palace; whose lofty entablatures, by a kind of looming, are seen with magical effect from every part of the union.

It is marvellous to see with what lustre the 'seals of office glitter in the eyes' of the good people of these United States. The whole world furnishes no parallel case. The number of state and federal officers is so great, as to awaken political aspirations on our entire male population; and some of them are invested with sufficient dignity, power, and patronage, to excite the most intense emulation—I ought rather to say, the angriest rivalry. Thus are the genius and ambition of the nation drawn into the race of political glory. But for this, the law would scarcely exert stronger attractions upon the talent of the country, than medicine; for its own intrinsic rewards are not of a higher order, as the problems which it offers, do not require for their solution, a greater amount of intellect, than the practice of medicine. How long this will continue to be the case, it is difficult to foresee; but as our population is progressive, while the number of political offices is nearly stationary, we may hope that the time is not distant, when the proportion of talented youth who are dedicated to the study of physic, will be much greater than at present.

The consequences of this deficiency of talent in the profession, are of serious import to the science and to the people at large. It is unquestionably one of the causes, which retard the progress of discovery and improvement. Of the thousands who annually go forth with diplomas or licenses, or without either, to engage in the practical duties of the profession, very few ever contribute a single new fact to its archives, or communicate an impulse to the minutest wheel in its complicated machinery. Acting on the precepts of others, they may, it is true, do some good, but they also do much harm; while to the great work of revising and correcting the principles of the science, they are of course utterly incompetent.

But this incompetency is not the effect of inferior *talents* only, it results perhaps in an equal degree, from want of education and mental discipline, on the extent and causes of which I shall now proceed to speak.

Although medicine is ranked with the *learned* professions, not a few of its professors are signally deficient in learning.* This is the case not only in the Western states, where for obvious reasons it might be expected; but in almost every part of the union, with the exception of some of our large cities. Writing as I do for practical effect, and to promote *reform*, I am constrained to say, that even at this late period, the profession abounds in students and practitioners, who are radically defective in spelling, grammar, etymology, geography, arithmetic, and, I might add book-keeping, but that they generally apply themselves to the study of that important

*Of course, on this occasion, I expect the reader to understand the word *professor* as synonymous with *practitioner*, and not as referring to public teachers; whose commissions must be regarded as evidence of learning, should other proofs *happen* now and then to be wanting.

branch with a diligence which supplies the want of early opportunities. Grammar and spelling especially, (to use the language in which I once heard a physician speak of the circulation of the blood,) appear to be among the '*secret arcanums of nature which Dr. Hamilton said never would be found out.*' Nothing is more common than to commit gross violations of both, in the directions which we write for our patients; and, what is still more humbling to the pride of the profession, not a few of us never learn to spell the names, either of the medicines which we administer, or the diseases which we cure. Was this confined to unauthorized members of the profession, it would be an affair of little magnitude; but extending to many of the graduates, of *all* our Universities, it calls for unreserved exposure and unqualified reprehension. Before the Revolution, the schools of the Colonies were generally bad, and till lately those of the West were not fitted to impart a good elementary education; but at present they are so improved, as to leave no excuse, for the literary ignorance which disgraces the profession. The taint, however, is hereditary, and may yet run through several generations, unless the authoritative members of the faculty can be moved to unite their prescriptions upon it. It would certainly not be unreasonable to require, that every youth who aspires to connect himself with a liberal pursuit, should first learn how to spell and write his mother tongue with as much accuracy as a country schoolmaster; if either his genius or misfortunes preclude such acquirements, he had better take to some calling which does not demand them.

On reading the foregoing sentences to a friend, I found him sceptical as to their accuracy; which leads me to repeat, that *I* am entirely convinced of it. Nothing could be further from my heart, than a desire to disparage the character of a profession, to which, 'man and boy,' I have been attached for nearly thirty years; and to the advancement of which my humble exertions have been devoted for most of that period. During the time I have learned something of the literary and professional acquirements, of so many students and physicians, in and of, various parts of the Union, that I

cannot be mistaken in asserting, that the majority of the profession in America, are deficient in common school learning. If such be the fact, it should not be considered libellous to publish it, especially when done by one who claims no exemption from the imperfections which he deplores. So long as we 'measure ourselves by ourselves and compare ourselves among ourselves' we are not likely either to perceive or supply our defects. There can be no true reformation without a consciousness of its necessity; and if these remarks should contribute but in the slightest degree to excite it, I shall submit cheerfully to the odium which they may bring upon me, from those who find recrimination more convenient than improvement.

But is the education which our common schools confer, a sufficient preparation for the study of medicine? It certainly is not. To a familiar acquaintance with the branches which have been enumerated, the intended student of medicine, should add a competent knowledge of the elements of physical geography, general history, the art of composition, algebra, geometry, and mechanics. If these acquirements are not made before he enters on his professional studies, he will most probably remain without them through his whole life; the effects of which will be sufficiently obvious to others, if not felt by himself. After what has been said, concerning our deficiencies in the rudiments of learning, it will scarcely be supposed, that our acquaintance with the sciences of this second group, is such as to constitute a suitable introduction to medical studies. In truth, most of us live and die in utter ignorance of them. There *was* a time, when this ignorance, particularly of mechanical philosophy, would have been thought fatal to the success of a student of medicine. Our science was then, held to be a branch of general physics; and the laws of the living system, a mere modification of those which govern the operations of dead matter. I would be among the last to desire the revival of these exploded errors; but that we have passed from one extreme to another, seems to me an unquestionable fact. We cannot explain the phenomena of living bodies, *by* the principles of natural philosophy, but at the same time are unable to comprehend them, without the aid of those principles. The functions of seeing, hearing, locomotion, respiration, and the circulation of the blood, can no more be understood without an acquaintance with the laws of natural philosophy, than the movements of the atmosphere or the heavenly bodies; but their agency in the two cases is widely different. In the movements of the universe, we behold *only* the influence of these principles; but in the functions of organized beings, they are subordinate to a vital power; the laws of which constitute the science of life, or physiology. Thus organized bodies present us with a case, in which the general laws of matter are not repealed, but subjected to modification. Among all the works of God, we meet with no others which present such a great assemblage of agencies;—so diversified, yet co-operative—so admirably balanced—so harmonious, though complex and apparently involved—so productive of striking and beautiful forms! The human system is, indeed, the great mystery of creation, offering problems of matchless intricacy, and shrouded from human observation, by a veil which none should attempt to draw aside, without deep and varied preparation.

Suppose, what is not the fact, that to common-school attainments, our students added the first principles of mathematics, and the other sciences, constituting an academical course, would they *then* be properly qualified? I again answer they would not; and this brings us to the consideration of the learned languages, as an introduction to professional studies. I shall not attempt to travel over the whole ground.

The United States are, perhaps, the only civilized country in modern times, where it has been seriously doubted, whether the languages and literature of the ancients, should make a part of the studies of professional men. Of the various causes which have combined to suggest this question, one of the most operative is the spirit of liberal inquiry, which originated, and is cherished by, our free institutions. No people are so unshackled by prejudices and precedents; none so excursive; none so experimental, as the American. If they do not 'try all things, and hold fast to that which is good,' they try many, and are strongly disposed to fix upon something new.

Another cause contributing to excite the same inquiry, is the successful acquisition of business by physicians, who lived and died in ignorance of Greek and Latin. With such examples before us, it was natural to ask, whether the study of the dead languages should be regarded as indispensable, or even beneficial, to the candidate for the honors of the profession; and not a few have, at all times, been ready to answer in the negative. In this inquiry there has been much to lead us astray.

Our forefathers, (most of whom were illiterate) emigrated to a forest, which it has been the occupation of their sons to subdue. In the prosecution of this Herculean task, and the subsequent establishment of institutions—political, social, and literary, they frequently experienced a want of appropriate means, and were compelled by the exigencies of their novel and trying situation, to think and act with originality. Hence arose a feeling of self reliance; a spirit of independence; a disregard of ancient customs; to which we may in a great degree ascribe that indifference to the languages and learning of antiquity, which characterizes the majority of our citizens.

Thus physical circumstances have, indirectly, exercised a mastery over moral causes, and given a deflection to our European character, which promises to become permanent. Moral causes, however, have contributed to the same effect. In migrating from the old world, our ancestors took leave of the institutions devoted to classical instruction; and hence a generation, of necessity, grew up in comparative ignorance. It would be in vain to hope, that a due respect for the learned languages, or even a conviction of their utility, could survive such a transition; and hence we find, that in the United States, a want of acquaintance with them, has been no serious obstacle to the attainment of high *relative* distinction, in any of the pursuits of society. How long this will be the case, it is not easy foresee. A perception of their value appears to be returning; but I cannot suppose, that they will ever attain to the rank which they hold in the estimation of our elder brethren of Europe.

Meanwhile, it is the duty of those who can exercise any control over public sentiment in this respect, to exert themselves; and, if all who are interested in the dignity of the medical profession, could be brought to unite their efforts in favor of a more classical preparation of young men designed for the study, it cannot be doubted, that much might be accomplished, even in a single generation.

A physician who is ignorant of the Latin and Greek languages, whatever may be his genius and professional skill, must, to the eye of sound scholarship, appear defective and uncultivated. For more than two thousand years, these languages, especially the former, were the vehicles of all medical knowledge, except the little contributed by the Arabians; and, till within a century, our professional ancestors wrote, and prescribed, and thought, and lectured, in Latin. It was, indeed, to the profession a universal language; affording the means of an easy and accurate correspondence, among all the schools and physicians of Europe. Even down to the present time, the lectures in most of the Italian and German Universities, are delivered in Latin; while the examination of candidates, in many others, is conducted in the same language. Thus it has had a most protracted and intimate companionship with medicine; to the nomenclature of which it has freely lent its opulent vocabulary. Many of its words, no doubt, as well as those drawn from the dialects of Greece, are intended to convey, in their new situation, ideas materially different from their vernacular import; but in attempting to understand even these, the student is greatly assisted by an acquaintance with their primitive significations. With this knowledge of our dependence on the languages and literature of the ancients, to deny that the study of them must be beneficial, is scarcely less absurd, than to affirm, on the other hand, that every classical scholar is of necessity a physician.

But a thorough course of preparatory learning is useful, in more ways than one. It establishes early habits of application; generates a love of knowledge; trains the faculties; and inspires that firmness of purpose, which prevents him who puts his hand to the plough, from looking back. These are the cardinal virtues of a student; and they are in a great degree the effect of cultivation. We look instinctively at the grand and beautiful aspects of nature, but this is poetry, not philosophy. A poet delineates the surface, a philosopher decomposes the substance of things. One is born, the other called to his vocation. Education never made a great poet, nor nature a good philosopher. He is essentially the product of art. Toil is his destiny. He must sink a deep shaft, and draw up his treasures from below. Therefore he should be strengthened by timely and active effort. He must be inured to labour, and acquire adroitness in its performance. Hence he should begin early, for then only can suitable habits be formed. As well might he attempt, at twenty, to learn fencing, dancing, or a delicate and difficult art, as to commence at that age, without previous study, a course of philosophy. In both cases his awkwardness gives birth to the most discouraging failures. He feels, incessantly, the want of that strength and discipline, which are the offspring of practice, and are derived from it only. I would admonish parents to consider this subject anxiously and deeply. Many who place their sons to the study of medicine, are themselves illiterate, and do not apprehend the importance of early intellectual discipline. They transfer the objects of their care, from the plough to the doctor's shop; and require them to exchange the recipies of agriculture for those of pharmacy. Of the multitude, a few, by the force of an irrepressible genius, may rise to eminence; but the majority must lag forever in the under walks of the profession. Not having learned to climb, when the art might have been acquired, they may assault, but cannot scale the rugged steeps of science.

It is sorrowful to see, what every physician must have seen, the exertions of a generous young man consigned to the study of medicine, with a mind untutored and unstored;—to witness his ill directed efforts—strong but comparatively unavailing; his fitful application; his embarrassments under every difficulty; his disappointments and despondency; above all his mortification, from a consciousness of superior abilities united with a perpetual conviction of inferior progress. No devotedness to study, no intensity of ambition, no energy of intellect, not the whole combined, can make such an one what he would have been with early culture, nor raise him to the standard erected by his own vivid imagination. He may satisfy his friends, but must himself remain dissatisfied and unhappy.

But let us turn to a more common case. Suppose the youth as deficient in strength, as he is in discipline of mind;—what will then be his progress? To answer with a paradox, it will be *no* progress. There can be no advancement, when both nature and art array themselves in opposition. The first lessons of science, are to such a person, a 'sealed book;' which, like the worm, he may perforate, but can never open. Or, to use a more national figure, he is a militia man without courage or discipline, when one, at least, is indispensable to success. He cannot take captive, the vanguard of postulates, axioms, and definitions which lie in his way; and of course the citadel remains permanently hidden from his dim and uneducated vision. If dull young men *must* be apprenticed to medicine, common sense dictates, and common justice to them and society requires, that the pedagogue should begin his drillings early; for dunces are neither plastic nor acquisitive, and where the tree of knowledge grows slowly, it should not be planted late.

With these remarks I close this branch of our subject. If I have spoken freely, it has been as a friend, not an enemy of the profession. I have not written from the recesses of a cloister, but in the midst of society. Observation and personal experience have dictated every sentence, and afforded me satisfactory evidence of its truth. For this, moreover, I might appeal to all my enlightened brethren of the United States. Not one of them could venture to gainsay, what has been asserted; nor commend the apathy which connives at the errors and abuses, which have been exposed. Let *them* begin the good work of reformation, and society will come quickly to their aid.

